# The role of attachment and personality traits in choosing opiate addiction replacement therapy

**DOI:** 10.1038/s41598-024-65695-w

**Published:** 2024-06-25

**Authors:** Alena Gizdic, Vesna Antičević, Igna Brajević-Gizdić

**Affiliations:** 1 University Department of Health Studies, University of Split, Split, Croatia; 2 Department of Psychology, Vanderbilt University, Nashville, TN USA; 3 Teaching Institute of Public Health, County of Split Dalmatia, Service of Mental Health, Split, Croatia

**Keywords:** Psychology, Health care, Medical research

## Abstract

Contemporary medical approaches for opioid addiction often include medication-assisted therapy, utilizing methadone and buprenorphine. However, factors influencing patient preferences for starting buprenorphine or methadone therapy are poorly understood. This study aims to explore whether variances in personality traits and attachment styles are related to treatment preferences among individuals undergoing buprenorphine and methadone maintenance therapies. 300 participants completed the Big Five Questionnaire for personality traits and sub-dimensions and the Experiences in Close Relationship Scale for assessing attachment styles. The results indicated that patients with higher levels of Dynamism, Conscientiousness, and Perseverance personality traits were more likely to choose buprenorphine over methadone for achieving and maintaining abstinence. Although attachment styles showed a greater ability to differentiate between groups compared to personality traits, the differences were not significant. However, Conscientiousness stood out for its high discriminant validity, suggesting that scores in this personality dimension could significantly distinguish between groups, with individuals in the buprenorphine group showing higher levels of Conscientiousness compared to the methadone group. The study suggests a partial association between individuals' preference for abstinence therapy and their personality traits. These findings could be considered useful indicators when choosing maintenance therapy to help opiate-addicted patients achieve and maintain abstinence.

## Introduction

Substance addiction has been conceptualized as a brain disease, substantiated by the alterations induced by drugs in the structure and functionality of the brain^1^. Characterized as a chronic, recurrent disease, addiction is proposed to arise from the interaction between the organism and the drug, driving behavioral and other changes driven by an irresistible internal compulsion to continue drug intake, despite awareness of harmful consequences (loss of control)^2^. As such, etiological models, including the biopsychosocial model of addiction^3^, conceptualize addiction as a multifaced disorder, wherein this internal compulsion is attributed not only to biological factors but also to psychological and social factors^3^. These psychosocial factors encompass individual psychological traits, such as personality and coping mechanisms, as well as social determinants such as environmental influences, socioeconomic status, and cultural norms^4^. Together, these components interact to shape an individual's susceptibility to addiction, the development of addictive behaviors, and the effectiveness of prevention and treatment strategies^5,6^. Contemporary medical practices for addressing opioid addiction frequently involve the application of medication-assisted therapy, notably utilizing methadone and buprenorphine, with the aim of managing addictive behaviors and facilitating the cessation of opiate use^7^. Methadone (MET), functioning as a full µ-opioid receptor agonist, engages with the brain's receptors identical to those targeted by opioids such as heroin or prescription pain medications, whereas buprenorphine (BUP), as a partial opioid agonist, attaches to opioid receptors exhibiting ceiling effect, meaning that beyond a specific dosage, it does not impart further effects, thereby diminishing the potential for misuse^8,9^. The literature underscores the importance of methadone and buprenorphine in alleviating withdrawal symptoms and cravings by acting on opioid receptors^10,11^. Notably, some patients prefer buprenorphine over methadone, finding it more useful and effective in treating opioid dependence, including the blockage of cravings^12^.

However, collaborative therapy decisions involve both the psychiatrist and the patient, with the choice of a specific therapy setting contingent upon various factors, including but not limited to the patient’s health status, medication availability, potential side effects, risk of abuse and overdose, cost considerations, and long-term effects^13,14^. Despite these considerations, limited knowledge exists regarding other decisive factors influencing patients' preference for initiating buprenorphine or methadone therapy. One such factor that may influence the preference of medication-based therapy is the individual's traits^15,16^. So far, existing research underscores the significant variability in personality traits among individuals with different substance use tendencies^17–19^. Individuals exhibiting elevated levels of neuroticism and high extraversion are more inclined towards the usage of cocaine/crack, heroin, and stimulants; heightened openness to experience demonstrates an association with cannabis use, while low levels of agreeableness links to cocaine/crack use and the illicit consumption of opioids^20–22^. Given the intricate nature of these associations, it is plausible to argue that personality traits may also play a significant role in shaping patients' inclination toward the choice of medication-based therapy for opioid addiction.

Another factor that offers a valuable framework for comprehending the origins of addictions and may be influencing the preference for medication-based therapies is the attachment style^23^. Attachment theory posits that early-life interactions with caregivers promotes the development of mental representation of self and others, as well as support systems received during vulnerable moments^24^. For instance, signaling distress and seeking proximity to a health care provider as well as negotiating for therapy in times of illness is likely to be expressed in a way that is consistent with attachment characteristics^25^, particularly in individuals with Substance Use Disorder (SUD)^26^. Although the literature regarding attachment styles and patients’ preference of methadone or buprenorphine therapy option is lacking, there are extensive links between attachment and substance dependency^27,28^, specifically examining highly insecure attachment patterns indicating divergent developmental trajectories among various groups of substance abusers. Particularly, the prevalence of a fearful–avoidant attachment pattern emerges prominently among individuals with heroin addiction^29^.

Given that addiction is widely recognized as a chronic, relapsing disease, and opioid addiction presents as a particularly challenging chronic condition, exploring the association between personality traits, attachment style, and treatment preference for buprenorphine and methadone therapies holds significant promise in optimizing addiction treatment outcomes. The provision of substitution therapy, such as buprenorphine and methadone, plays a crucial role in harm reduction approaches for individuals with addiction, facilitating early stabilization and functional improvement in familial and work environments^12,30,31^. Furthermore, by aligning treatment preferences with patients' personality traits and attachment styles, clinicians can increase treatment retention and satisfaction, as well as decrease the risks associated with illegal "self-medication," involvement in criminal activities, and exposure to additional health risks^32,33^. This can be achieved by offering therapies that resonate with patients' psychological and emotional needs, thereby reducing dropout rates and enhancing long-term recovery prospects^34^.

### The present study

Research addressing the distinct personality traits and attachment styles of individuals undergoing medication-based therapy for opioid addiction, particularly those receiving methadone and buprenorphine therapy, is currently lacking. Therefore, our study seeks to: (a) elucidate variations in personality traits and attachment styles within the subset of individuals undergoing buprenorphine and methadone maintenance therapy, and (b) investigate whether these individuals differ in personality traits and attachment styles depending on their choice of the maintenance therapy. Given the empirical evidence on personality traits and insecure attachment style within the context of addiction, we expect that the choice of methadone or buprenorphine therapy by individuals with opioid addiction will be influenced, in part, by their personality traits and attachment patterns. We also expect that individuals opting for buprenorphine maintenance therapy (considered more useful and effective in treating opioid dependence) will exhibit a less pronounced insecure attachment style and more favorable personality traits, reflected by higher scores on five major dimensions of personality, in comparison to their counterparts receiving methadone maintenance therapy.

## Methods

### Participants and procedures

The research was initiated by a qualified psychiatrist at the Service for Mental Health, Addiction Prevention, and Outpatient Treatment in Split, Croatia. The data were collected by a trained researcher during regular outpatient consultation times. Before starting the survey, participants were informed about the objectives and procedure of the research study, and they acknowledged that their participation was voluntary and confidential. All participants were provided with informed consent to take part in the study, agreeing to have their data used for analysis. To prevent coercion or undue influence, regular monitoring of psychiatrist-patient dynamics was maintained throughout the study. The study included 300 individuals with heroin addiction, divided evenly into two distinct groups. Group 1 consisted of 150 individuals receiving methadone maintenance therapy for heroin addiction, while Group 2 comprised of 150 individuals undergoing buprenorphine maintenance therapy. Inclusion criteria were defined as follows: a diagnosis of opioid dependence (ICD-10 Code F11.2)^35^, a documented 12-month abstinence from heroin as indicated in medical records maintained by the mental health support service, a negative result in the urine drug test taken prior the study assessment, and a positive test for the prescribed maintenance therapy on the day of administering the measurement instruments. This positive result was determined through the use of the panel drug test for detecting consumption of opioids such as heroin, morphine, and codeine present in a urine sample at a concentration of at least 300 ng/ml (MOP), for detecting buprenorphine at a concentration of 10 ng/ml (BUP) and for detecting methadone (Heptanone, substitution therapy) at a concentration of 300 ng/ml (MTD). Exclusion criteria included failure to register with the Croatian National Register of Treated Psychoactive Drug Addicts at the Croatian Institute of Public Health and the presence of cognitive impairment or intellectual disability.

Ethical approval for the study was obtained from the Ethics Committee of the School of Medicine, University of Zagreb, aligning with research activities conducted at the Teaching Institute of Public Health, County of Split Dalmatia, Service of Mental Health, one of the main educational and research bases within the University, operating under valid permits of the competent University authorities. All participants provided written informed consent before participating.

### Measures

The Big Five Questionnaire (BFQ) was utilized to assess the personality traits^27^, The BFQ, aligned with the five-factor model of personality, examines five principal dimensions, each encompassing two sub-dimensions: Energy (Dynamism and Dominance), Agreeableness (Cooperativeness/Empathy and Warmth/Friendliness), Conscientiousness (Scrupulousness and Perseverance), Emotional Stability (Emotional Control and Impulse Control), and Openness (Cultural Openness and Openness to Experience) and a Lie scale. The inventory contains 132 statements that are distributed across the dimensions and sub-dimensions. Each dimension includes 24 statements, while sub-dimensions consist of 12 statements each, scored on a 5-point scale (1 = 'not at all true'; 5 = 'exactly true'). Total scores are derived by summing the scores for specific items within each sub-dimension, facilitating the computation of total scores for the BFQ dimensions^36^.

To evaluate distinct dimensions of attachment within friendships, a shortened version of the Experiences in Close Relationships Scale (ECR) was employed. The scale had been validated among a Croatian population^37^, containing identical psychometric characteristics to the original scale^38^, (detailed in^37^). The scale, comprising 18 statements, evaluates individuals' feelings, thoughts, and behavior in close relationships. Participants rate each item on a 7-point scale, ranging from 'strongly disagree' to 'strongly agree,' based on how well it reflects their own experiences with friends in general. The scale consists of two subscales, Anxiety and Avoidance, each comprising 9 items, demonstrating high reliability (Cronbach’s alpha: 0.86 for Avoidance, 0.83 for Anxiety). The orthogonal subscales strongly correlate with their underlying factors (r = 0.08).

### Data analysis

The characteristics of the studied groups were described using descriptive parameters. Pearson’s correlation coefficient was used to explore associations between personality traits and attachment styles, providing insights into the relationships within the buprenorphine and methadone groups. To examine significant differences between the two groups including demographic characteristics as well as group means pertaining to personality traits and attachment styles, T-test and χ^2^ test scores were employed. Finally, to examine whether two groups of participants differ on a set of variables and determine which variables contribute most to group separation, discriminant analyses were employed. Initially, the potential differentiation of the two groups was investigated based on their scores on the BFQ questionnaire and subsequently on the ECR questionnaire. Three discriminant analyses were conducted, one for each of the three groups of variables: traits of the five-factor personality model (5 variables) and individual sub-dimensions of the traits of the five-factor personality model (10 variables), followed by dimensions of attachment (2 variables).

### Ethics approval

All procedures followed were in accordance with the ethical standards of the responsible committee on human experimentation (institutional and national) and with the Helsinki Declaration of 1975, as revised in 2000 (5). Ethical clearance for the study was obtained from the Ethics Committee of the School of Medicine, University of Zagreb.

## Results

The total sample included 300 participants. 150 participants belonged to the buprenorphine group (mean age = 38.6; SD = 5.9; 86% male) and 150 participants belonged to the methadone group (mean age = 41.5; SD = 7.4; 79% male). There was a significant difference in age between the two groups (χ^2^ = 3.681, p < 0.001). The methadone group had a higher average age compared to the buprenorphine group. Additionally, there was a significant difference in marital status between the two groups (χ^2^ = 10.587, p = 0.032). Specifically, the buprenorphine group had a lower percentage of patients who are not married compared to the methadone group. (See descriptive statistics in Table 1).

**Table 1:** Descriptive information (N = 300).

	Groups		χ^2^	p*
	Buprenorphine (N = 150)	Methadone (N = 150)		
Therapy (mg)	M = 11.31, SD = 7.496	M = 79.97, SD = 35.457	23.201	< 0.001*
Age	M = 38.6, SD = 5.9	M = 41.5, SD = 7.4	3.681	< 0.001*

	N (%)	N (%)		
Gender				
Male	129 (86%)	118 (79%)	2.292	0.130
Female	21 (14%)	32 (21%)		
Education				
Bachelor's degree or below	118 (78.8%)	124 (82.7%)	2.152	0.112
Marital status				
Not married	74 (49.3%)	84 (56%)		
Married	40 (26.6%)	29 (19.4%)		
Divorced	17 (11.3%)	27 (18%)	10.587	0.032*
Cohabiting	17 (11.3%	6 (4%)		
Widow	2 (1.3%)	4 (2.6%)		
Parenting	66 (44%)	56 (37%)	1.338	0.247

*N* number, *M* mean, *SD* standard deviation; χ^2^ = chi square.

*p-level of significance (5%).

The bivariate correlations between personality traits and dimensions of attachment in both groups are presented in Supplemental Table 1. According to Cohen^39^, effect size of 0.10 is small, 0.30 is medium, and 0.50 is a large effect size.

### Differences in personality traits and attachment styles

While the majority of sub-dimensions of the BFQ exhibited a tendency towards higher mean values in the buprenorphine maintenance therapy group, statistically significant distinctions were observed between the two groups in Dynamism (t = 2.020; p = 0.044), Conscientiousness (t = 2.759; p = 0.006), and Perseverance (t = 3.589; p < 0.001). The difference in Agreeableness approached statistical significance (t = 1.943; p = 0.053). In all instances, the average score was notably higher in the buprenorphine maintenance therapy group (Table 2). No statistically significant differences were identified between the groups concerning scores on either dimension of attachment-related anxiety or avoidance (Table 2).

**Table 2:** Differences in personality traits and attachment styles of individuals with opiate addiction treated by methadone and buprenorphine maintenance therapy.

Personality traits (BFQ)	Groups		t	p
	Buprenorphine	Methadone		
	M ± SD	M ± SD		
Energy	49.5 ± 7.62	47.7 ± 9.04	1.823	0.069
Dynamism	**49.2 ± 8.90**	47.1 ± 9.22	2.020	0.044*
Dominance	50.0 ± 07.91	49.0 ± 8.59	0.993	0.322
Agreeableness	50.9 ± 9.05	49.0 ± 7.91	1.943	**0.053**^**T**^
Cooperativeness/Empathy	51.6 ± 9.44	49.8 ± 8.80	1.758	0.08
Warmth/Friendliness	50.2 ± 8.93	48.7 ± 8.53	1.435	0.152
Conscientiousness	**46.3 ± 9.03**	43.4 ± 9.05	2.759	0.006*
Scrupulousness	48.8 ± 8.71	47.4 ± 8.79	1.359	0.175
Perseverance	**44.8 ± 8.85**	41.1 ± 8.94	3.589	< 0.001*
Emotional stability	48.7 ± 9.40	47.7 ± 8.74	0.973	0.331
Emotional control	48.8 ± 9.92	47.8 ± 9.21	0.910	0.363
Impulse control	48.9 ± 8.56	48.0 ± 8.50	0.873	0.383
Mental openness	50.9 ± 9.66	49.5 ± 9.99	1.216	0.225
Cultural openness	51.4 ± 9.68	50.9 ± 10.20	0.488	0.626
Openness to experience	50.2 ± 8.79	48.3 ± 9.03	1.840	0.67
Lie scale	47.5 ± 9.79	47.6 ± 9.78	-0.077	0.939

Attachment (ECR)	M ± SD	M ± SD	t	p
Anxiety	26.30 ± 9.74	27.54 ± 9.87	-1.095	0.274
Avoidance	27.21 ± 7.71	27.74 ± 8.68	-0.555	0.579

M-mean; SD- standard deviation; t – test; *p-level of significance (5%); ^T^ trend p < 1.0; BFQ = The Big Five Questionnaire; ECR = Experiences in Close Relationships Scale.

### Discriminant analysis of group differentiation in the five-factor model of personality and attachment styles

The discriminant function initially classifying participants as treated with buprenorphine or methadone based on the five major personality traits of the five-factor personality model was not significant (χ^2^ = 10.453; p = 0.063). However, a canonical correlation between group membership and the obtained discriminant function was 0.186, with the Conscientiousness trait loading most strongly on the discriminant function. The data revealed that 56% of participants were correctly classified by the discriminant function (i.e., if groups were not known a priori, 56% of participants would have been correctly classified based on their scores on the five-factor model). The accuracy was 6% better than the maximum using random guessing (which yielded a 50% overall classification accuracy). The accuracy was slightly better for the methadone maintenance therapy group than for the buprenorphine group. The values and coefficients of the test and the results of the discriminant analysis are displayed in Table 3.

**Table 3:** Discriminant analysis of five major BFQ personality traits in buprenorphine and methadone group.

	Eigenvalue	Canonical correlation	Wilks' Lambda value	χ^2^	p
BFQ-5	0.036	0.186	0.965	10.453	0.063

Group	Predicting group affiliation				
	Buprenorphine	Methadone			
Buprenorphine N (%)	82 (54.7)	68 (45.3)			
Methadone N (%)	64 (42.7)	86 (57.3)			

Canonical correlation coefficient and eigenvalue; χ^2^- chi-square test; p-level of significance (5%).

Number and percentage of correctly identified participants based on the five major personality traits of the Big Five Questionnaire (BFQ) results–56.0% correctly classified.

Additionally, when classifying participants as treated with buprenorphine or methadone based on the sub-dimensions of the five-factor personality model, the discriminant analysis approached significance levels (χ^2^ = 18.06; p = 0.054). A canonical correlation between group membership and the obtained discriminant function was 0.244, with the Perseverance sub-dimension (corresponding to the major Conscientiousness personality trait) loading most strongly on the discriminant function. The data revealed that 59.3% of participants were correctly classified by the discriminant function, indicating 9.3% better accuracy than the maximum using random guessing. Thus, the accuracy was again slightly better for the methadone maintenance therapy group than for the buprenorphine group. The values and coefficients of the test and the results of the discriminant analysis are displayed in Table 4.

**Table 4:** Discriminant analysis of sub-dimensions of BFQ personality traits in buprenorphine and methadone group.

	Eigenvalue	Canonical correlation	Wilks' Lambda value	χ^2^	p
BFQ-10	0.064	0.244	0.940	18.059	0.054

Group	Predicting group affiliation				
	Buprenorphine	Methadone			
Buprenorphine N (%)	85 (56.7)	65 (43.3)			
Methadone N (%)	57 (38)	86 (62)			

Canonical correlation coefficient and eigenvalue; χ^2^- chi-square test; p-level of significance (5%).

Number and percentage of correctly identified participants based on the personality sub-dimensions of Big Five Questionnaire (BFQ) results–59.3% correctly classified.

Lastly, the discriminant function classifying participants as treated with buprenorphine or methadone based on the anxiety and avoidant attachment style significantly distinguishes the group on buprenorphine from the group on methadone, with a canonical correlation of 0.352. The obtained discriminant function allows us to correctly classify 67.0% of the participants based on the results of anxiety and attachment dimensions. This is 17% better than random guessing, which, when distinguishing between two groups, is 50% overall classification accuracy. The values and coefficients of the test and the results of the discriminant analysis are displayed in Table 5.

**Table 5:** Discriminant analysis of anxiety and avoidant attachment dimensions of ECR scale in buprenorphine and methadone group.

	Eigenvalue	Canonical correlation	Wilks' Lambda value	χ^2^	p
ECR	0.141	0.352	0.876	38.2	< 0.05*
	Predicting group affiliation				

Group	Buprenorphine	Methadone			
Buprenorphine N (%)	96 (64)	54 (36)			
Methadone N (%)	45 (30)	105 (70)			

Canonical correlation coefficient and eigenvalue; χ^2^- chi-square test; p-level of significance (5%).

Number and percentage of correctly identified participants based on based on the Experiences in Close Relationships Scale (ECR) results–67% correctly classified.

## Discussion

This study investigated personality traits and attachment styles in individuals undergoing buprenorphine and methadone maintenance therapy. It aimed to explore differences in these traits based on the selected maintenance therapy, considering the influence of personality and attachment patterns on therapy choices in substance addiction. The findings revealed that individuals on buprenorphine maintenance therapy showed higher levels of Conscientiousness and slightly elevated Agreeableness compared to those on methadone therapy. Additionally, buprenorphine recipients exhibited significantly increased levels of Dynamism and Perseverance. Concerning the attachment dimension, no statistically significant distinction was found between the two groups.

The Conscientiousness dimension within the Big Five Questionnaire (BFQ) stands out for its high discriminant validity, indicating that scores in this personality dimension can reveal significant differences between groups. Conscientiousness, reflecting self-regulation and self-control, includes traits such as reflexivity, orderliness, precision, and persistence^36^. Notably, individuals opting for buprenorphine therapy showed elevated Conscientiousness levels, suggesting a preference for this treatment, while those with lower Conscientiousness tended to select methadone.

Despite the statistical approach of the discriminant function based on the five-factor model of personality, the classification accuracy marginally favored the methadone maintenance group. The discriminant analysis results indicated that just over half of individuals undergoing buprenorphine therapy were correctly classified, with a corresponding classification accuracy of 57.3% for the methadone group. The group prediction classifier relying on BFQ scores demonstrated slightly superior performance compared to random guessing, achieving an accuracy of 56%. While the BFQ exhibited some predictive validity, the modest improvement over random guessing emphasizes the ongoing need for research and refinement in comprehending the intricate relationship between personality traits and treatment choices.

Previous studies have depicted individuals with opioid addiction as commonly exhibiting traits such as anxiety, vulnerability, sensitivity, impulsivity, impatience, and irritability, often coupled with tendencies towards passivity, uncooperativeness, lack of empathy, and low levels of conscientiousness and perseverance^40,41^. Interestingly, those on buprenorphine therapy for opioid addiction diverge from these typical addiction profiles as the mentioned personality traits expressed in this group describe them as being active, energetic, and talkative, as well as demonstrating higher levels of conscientiousness, orderliness, precision, and perseverance compared to their counterparts on methadone, aligning with our initial hypothesis. Furthermore, these traits have been noted in non-drug users when compared to users^42^, which further aligns with the personality traits exhibited by participants undergoing buprenorphine therapy. Conversely, in a study of different groups of psychoactive substance users, the results showed that the profiles of cocaine/heroin users achieve very high vulnerability in the dimension of neuroticism (emotional instability), and very low competence and success as well as achievement orientation in the dimension of conscientiousness of the five-factor model of personality^43^. These personality traits better correspond to the characteristics of our participants on methadone therapy. It is thus possible that precisely individuals with opioid addiction exhibiting high Conscientiousness and Perseverance, coupled with proactive behavior, may be inclined to choose buprenorphine—a partial opioid receptor antagonist—in their pursuit of abstinence.

Conscientiousness as a personality trait appears to play a pivotal role in guiding individuals towards buprenorphine. Its partial opioid receptor antagonism potentially serves as a motivating factor for individuals to stabilize in abstinence quickly—aiming not just to avoid withdrawal crises, but to achieve absolute chemical control of addiction, thereby accepting the absence of relapse readily. Similarly, the heightened Dynamism and Perseverance observed in these individuals further influence their preference for buprenorphine therapy, as they may take responsibility for independent daily medication. These traits are integral to their everyday functioning and may influence their choice towards buprenorphine. Importantly, it is emphasized that their motivation is not driven by seeking the 'high' associated with methadone, as noted in previous studies^44^. Instead, these individuals seek a 'clear-headed' state, as highlighted in the work of Whelan and Remski^45^. Conversely, the fact that no significant difference in Emotional Stability and Mental Openness between the two groups of participants was found may indicate that they are equally sensitive and open to new experiences. It is possible that these characteristics may lead them into risky behavior and subsequently into addiction.

In terms of attachment dimensions and personality traits predicting the choice between buprenorphine and methadone therapy for opioid addiction, discriminant analysis results indicated that attachment styles exhibited superior discriminatory efficacy compared to personality traits. The predictive validity was notably elevated, particularly for the methadone group, where 70% of individuals were accurately classified. The classifier relying on attachment dimensions questionnaire scores demonstrated a predictive validity of 67%, marking a significant 17% improvement over random guessing.

Existing research consistently highlights attachment insecurity among individuals with diverse mental disorders, including addiction^24,46,47^. Notably, individuals with heroin addiction often display a general inclination toward insecure attachment, predominantly characterized by a fearful–avoidant attachment style^48,49^. Based on the aforementioned results, we assumed differences between groups of individuals with heroin addiction undergoing different types of substitution therapy in a way that individuals on buprenorphine have a more secure attachment style and are less insecurely attached compared to individuals on methadone maintenance therapy. However, despite this general inclination, the specific choice between buprenorphine and methadone treatment did not emerge as a distinctive factor depending on attachment styles in this particular cohort. The decision-making process for choosing opioid addiction therapy is complex, with attachment style representing just one facet within a broader context. While attachment style can influence interpersonal dynamics and mental health, its predictive power in determining the specific choice of therapy may be limited due to the multitude of factors considered by individuals and healthcare professionals in the treatment decision-making process^50^.

The strength of our current study underscores distinctions between individuals undergoing buprenorphine and methadone maintenance therapy concerning personality traits and attachment styles—a novel investigation, particularly in the context of Croatia. The research involved a substantial patient population receiving treatment from specialized physicians authorized to prescribe opioid substitution therapy, including psychiatrists, epidemiologists, and school and adolescent medicine doctors. These specialists underwent standardized training, mitigating potential biases in physician attitudes that might impact patients' choices between methadone and buprenorphine substitution therapy. However, the study has limitations, primarily its cross-sectional nature, which restricts inferences about the causal effects of therapy selection. Furthermore, while the observed differences in age and marital status between the buprenorphine and methadone treatment groups may offer valuable insights into the characteristics of the study population, understanding the complex relationships between demographic factors, treatment choice, and outcomes requires further investigation. Exploring potential confounding variables, such as socioeconomic status, comorbidities, or prior treatment history, in future studies can enhance our understanding of the nuanced factors influencing treatment decisions and outcomes. Additionally, the inability to ascertain whether disparities in personality traits predated substance use initiation or were instead associated with the therapy provided poses a common methodological challenge in studies on personality traits in individuals with opioid addiction. Nevertheless, this study marks the initiation of a quest for psychological tests and measurements aiming to expedite and enhance specialists' precision in selecting appropriate substitution therapies. The study's effort seeks to streamline the therapeutic process, fostering intervention, stabilization, and optimal functional outcomes for individuals grappling with addiction.

In sum, this study is the first to investigate the differences in personality traits and attachment styles between recipients of buprenorphine and methadone maintenance therapy among individuals with heroin addiction. The findings suggest that personality traits and attachment styles may serve as viable, objective, and quantifiable criteria for the judicious selection between methadone and buprenorphine therapy. If subsequent research substantiates the outcomes of this study, psychometric tests may emerge as valuable tools for formulating more precise guidelines in prescribing substitution therapy. Future research should explore additional psychological attributes, and investigate potential factors influencing the choices of substitution therapies among individuals with heroin addiction. This expanded focus would contribute to a more comprehensive understanding of the interplay of psychological elements in therapy selection, thus advancing the refinement of therapeutic interventions and informing evidence-based policies.

### Supplementary Information

Supplementary Table 1.

## Data Availability

The data that support the findings of this study are available on request from the corresponding author A.G. The data are not publicly available due to privacy or ethical restrictions.
