# Supplementary material for: The role of attachment and personality traits in choosing opiate addiction replacement therapy
Source: Sci Rep. 2024 Jun 25;14:14623. doi: 10.1038/s41598-024-65695-w (PMC11199502; doi:10.1038/s41598-024-65695-w)
Supplement: Supplementary file 1 — Supplementary Table 1. [file 41598_2024_65695_MOESM1_ESM.docx]

**The role of attachment and personality traits in choosing opiate addiction replacement therapy**

Alena Gizdic^1,2,*^, Vesna Antičević^1^, Igna Brajević-Gizdić^3^

**Supplemental Table 1. Pearson correlations between personality traits and dimensions of attachment**

|  | **BUPRENORPHINE** | | **METHADONE** | |
| --- | --- | --- | --- | --- |
|  | **Anxiety** | **Avoidance** | **Anxiety** | **Avoidance** |
| **Energy** | .019 | -.065 | .051 | -.078 |
| Dynamism | -.101 | -.133 | -.050 | -.121 |
| Dominance | .135 | .033 | .152 | -.002 |
| **Agreeableness** | .100 | -.060 | -.201* | -.207* |
| Cooperativeness/Empathy | .103 | -.009 | -.182* | -.231** |
| Warmth/Friendliness | .065 | -.082 | -.131 | -.102 |
| **Conscientiousness** | -.054 | -.013 | .011 | -.082 |
| Scrupulousness | .125 | .108 | .098 | -.082 |
| Perseverance | -.228** | -.141 | -.094 | -.100 |
| **Emotional Stability** | **-.318**** | -.157 | -.241** | -.134 |
| Emotional Control | **-.384**** | -.239** | -.257** | -.153 |
| Impulse Control | -.171* | -.023 | -.178* | -.088 |
| **Mental Openness** | -.166* | -.182* | .040 | -.079 |
| Cultural Openness | -.158 | -.139 | .083 | .019 |
| Openness to Experience | -.133 | -.179* | -.011 | -.175* |
| **Lie scale** | .186* | .079 | .073 | .135 |

** Correlation is significant at the 0.01 level (2-tailed).

* Correlation is significant at the 0.05 level (2-tailed).

*Note*2: Medium effect sizes in bold (.30).
